# Supplementary material for: Gaze-centered gating, reactivation, and reevaluation of economic value in orbitofrontal cortex
Source: Nat Commun. 2024 Jul 22;15:6163. doi: 10.1038/s41467-024-50214-2 (PMC11263430; doi:10.1038/s41467-024-50214-2)
Supplement: Supplementary file 1 — Supplementary Information [file 41467_2024_50214_MOESM1_ESM.pdf]

Supplementary Information

Gaze-centered gating, reactivation, and reevaluation of economic value in orbitofrontal cortex

Demetrio Ferro[1,2,*], Tyler Cash-Padgett[3], Maya Zhe Wang[3], Benjamin Y. Hayden[4], Rubén Moreno-Bote[1,2,5]

[1] Center for Brain and Cognition, Universitat Pompeu Fabra, 08002, Barcelona, ES;

[2] Department of Information and Communication Technologies, Universitat Pompeu Fabra, 08002, Barcelona, ES;

[3] Department of Neuroscience, Center for Magnetic Resonance Research, University of Minnesota, Minneapolis, MN55455, USA.

[4] Department of Neurosurgery, Baylor College of Medicine, Houston, TX, 77030.

[5] Serra Húnter Fellow Programme, Universitat Pompeu Fabra, Barcelona, Spain.

* corresponding author: demetrio.ferro@upf.edu

**Subject 1**				**Subject 2**			
**Area**	**Session**	**#cells**	**# trials**	**Area**	**Session**	**#cells**	**#trials**
13	1	51	643	11	1	18	1015
13	2	59	700	11	2	32	323
11	3	24	697	11	3	9	1084
11	4	29	603	11	4	26	906
**Total**		163	2643	**Total**		85	3328
**Mean±sem**		40.75±8.45	660.75±23.28	**Mean±sem**		21.25±4.99	832.00±173.58
**Subjects 1:2**		**Total**	**#cells**	248	**#trials**	5971	
		**Mean±sem**	**#cells**	31±5.85	**#trials**	746.38±87.29	

Supplementary Table ST1. Number of sessions, cells, and trials for the two subjects.

	first=L	***first=R***	***choice=L***	***choice=R***	***EV_L>EV_R***	***EV_L<EV_R***	***EV_L=EV_R***
**Subject 1**	1310<br>(49.56%)	1333<br>(50.44%)	1174<br>(44.42%)	1469<br>(55.58%)	1298<br>(49.11%)	1294<br>(48.96%)	51<br>(1.93%)
**Subject 2**	1676<br>(50.36%)	1652<br>(49.64%)	1790<br>(53.79%)	1538<br>(46.21%)	1660<br>(49.88%)	1593<br>(47.87%)	75<br>(2.25%)
**Total**	2986<br>(50.00%)	2985<br>(50.00%)	2964<br>(49.64%)	3007<br>(50.36%)	2958<br>(49.54%)	2887<br>(48.35%)	126<br>(2.11%)
offer1 LookL<br>***LookR***	**78.83%**<br>21.17%	18.43%<br>**81.57%**	**87.11%**<br>12.89%	**73.44%**<br>57.83%	**84.62%**<br>15.38%	**76.20%**<br>23.80%	**69.84%**<br>30.16%
delay1 LookL<br>***LookR***	44.78%<br>55.22%	35.51%<br>64.49%	**62.77%**<br>37.23%	46.65%<br>**53.35%**	**60.38%**<br>39.62%	49.07%<br>**50.93%**	49.21%<br>50.79%
offer2 LookL<br>***LookR***	18.18%<br>**81.82%**	**75.78%**<br>24.22%	32.32%<br>**67.68%**	10.31%<br>**89.69%**	30.34%<br>**69.66%**	12.05%<br>**87.95%**	19.84%<br>**80.16%**
delay2 LookL<br>***LookR***	43.30%<br>56.70%	41.34%<br>58.66%	**67.17%**<br>32.83%	35.11%<br>**64.89%**	**63.65%**<br>36.35%	38.32%<br>**61.68%**	48.41%<br>51.59%
choice LookL<br>***LookR***	49.30%<br>50.70%	50.25%<br>49.75%	**99.90%**<br>0.10%	0.17%<br>**99.83%**	**75.08%**<br>24.92%	23.56%<br>**76.44%**	54.76%<br>45.24%

offer1 LookL	**80.21%**	***delay1 LookL***	54.63%	***(Fig. 3F) offer1 LookL and delay1 LookL***<br>***offer1 LookL and delay1 LookR***	**47.95%**<br>32.26%
offer1 LookR	19.79%	***delay1 LookR***	45.37%	***(Supp. Fig. S5A) offer1 LookR and delay1 LookL***<br>***offer1 LookR and delay1 LookR***	6.68%<br>13.11%
offer2 LookL	21.20%	***delay2 LookL***	50.98%	***(Supp. Fig. S5B) offer2 LookL and delay2 LookL***<br>***offer2 LookL and delay2 LookR***	14.50%<br>6.70%
offer2 LookR	**78.80%**	***delay2 LookR***	49.02%	***(Fig. 3G) offer2 LookR and delay2 LookL***<br>***offer2 LookR and delay2 LookR***	36.48%<br>**42.32%**

Supplementary Table ST2. Number of trials (% fractions) for different task conditions. Fraction of trials for the two different screen sides (*LookL*/*LookR*) in main task epochs. Trials with EV_L=EV_R are cases where both offers were safe ($EV_L = EV_R = 1$). To capture meaningful patterns, offer presentation times and delay times (*offer 1 / delay 1 / offer 2 /delay2*) are cut to the last 200 ms of their duration. For *choice* time we used the first 440 ms of *choice-hold* epoch time allowing to capture almost all choices (with rare, 0.27% occasional misses). Trials are sorted with reference to first offer anchored on the left screen side (trials with first offer on right screen side are mirrored).

	Subject 1				Subject 2			
	session1	session2	session3	session4	session1	session2	session3	session4
$w_0^{(1)}$	2.57**	0.69 -	1.19 -	2.40*	-0.47 -	1.38 -	1.05 -	-0.53 -
$w_{1L}^{(1)}$	2.73***	2.30***	2.63***	4.19***	4.75***	2.17**	4.48***	2.97***
$w_{2L}^{(1)}$	1.68***	1.95***	2.00***	3.29***	3.35***	2.84***	3.90***	3.16***
$w_{3L}^{(1)}$	1.74 -	3.48 -	4.52*	15.17***	7.34**	12.01**	7.89**	8.10**
$w_{1R}^{(1)}$	1.99***	1.87***	2.24***	3.13***	4.17***	1.32*	2.33***	3.11***
$w_{2R}^{(1)}$	1.64***	2.04***	1.67***	3.83***	3.90***	3.22***	4.25***	3.13***
$w_{3R}^{(1)}$	-0.86 -	4.30 -	6.02*	13.94***	15.27***	15.42***	15.34***	8.94***
$w_0^{(2)}$	3.02***	1.37 -	1.12 -	-0.54 -	0.00 -	-1.28 -	0.07 -	-0.33 -
$w_{1L}^{(2)}$	2.86***	2.36***	3.05***	1.75***	5.06***	2.12***	3.35***	3.00***
$w_{2L}^{(2)}$	1.93***	2.33***	2.22***	2.21***	3.81***	2.72***	3.75***	2.40***
$w_{3L}^{(2)}$	-2.28 -	-0.89 -	3.22 -	7.92**	10.52***	9.58*	11.26***	5.72*
$w_{1R}^{(2)}$	1.53***	1.78***	2.60***	2.27***	5.20***	2.87***	3.16***	2.75***
$w_{2R}^{(2)}$	2.04***	1.91***	1.98***	1.69***	4.23***	3.41***	4.19***	2.37***
$w_{3R}^{(2)}$	-0.12 -	2.59 -	4.91 -	8.17***	9.47**	-0.71 -	13.95***	11.37***

Supplementary Table ST3. Weights for the logistic regression of choice and offer value, magnitude, and risk. The regression is applied for left offer (EV_L, m_L, σ_L^2) and right offer (EV_R, m_R, σ_R^2) as $\text{logit}(chR) = w_0 - w_{1L}EV_L - w_{2L}m_L - w_{3L}\sigma_L^2 + w_{1R}EV_R + w_{2R}m_R + w_{3R}\sigma_R^2$ in disjoint subsets $S^{(1)}$ and $S^{(2)}$, respectively denoted as $w_{\{1,2,3\}\{L,R\}}^{(1)}$ and $w_{\{1,2,3\}\{L,R\}}^{(2)}$. Note: EV range is $[0, 3]$; m range is $[0, 3]$; σ^2 range is $[0, 0.75\ (= 0.5 \cdot 0.5 \cdot 3)]$. The values could vary in the two subsets due to random sampling. Significance assessed via *F*-statistics, FDR corrected (- n.s., *p<0.05, **p<0.01, ***p<0.001).

	offer1	*delay1*	*offer2*	*delay2*	*re-fixate*	*choice-go*	*ch-hold*
all trials	β_0 0.04 -<br>β_1 1.74***	β_0 0.04 -<br>β_1 1.74***	β_0 0.04 -<br>β_1 1.74***	β_0 0.04 -<br>β_1 1.74***	β_0 0.04 -<br>β_1 1.74***	β_0 0.04 -<br>β_1 1.74***	β_0 0.04 -<br>β_1 1.74***
f_R > 0.75	β_0 0.57*<br>β_1 1.52***	β_0 0.49***<br>β_1 1.60***	β_0 0.33***<br>β_1 1.63***	β_0 0.62***<br>β_1 1.73***	β_0 0.31***<br>β_1 1.78***	β_0 0.31***<br>β_1 1.73***	β_0 7.73***<br>β_1 1.07 -
f_R < 0.25	β_0 -0.07 -<br>β_1 1.77***	β_0 -0.20***<br>β_1 1.79***	β_0 -0.82***<br>β_1 1.60***	β_0 -0.66***<br>β_1 1.54***	β_0 -0.18***<br>β_1 1.68***	β_0 -0.20***<br>β_1 1.67***	β_0 -6.98***<br>β_1 -0.14 -

Supplementary Table ST4. Weights for the logistic regression of choice and difference in EV of the two offers. Regression weights (β_0, β_1) and significance for results in Fig. 2A, assessed via *F*-statistics and FDR corrected (significance: - n.s., *p<0.05, **p<0.01, ***p<0.001).

	offer1	*delay1*	*offer2*	*delay2*	*re-fixate*	*choice-go*	*ch-hold*
all trials	β_0 0.16*** β_1 0.38***	β_0 0.18*** β_1 0.55***	β_0 -0.24*** β_1 0.83***	β_0 -0.22*** β_1 1.08***	β_0 -0.06* β_1 0.40***	β_0 0.05* β_1 0.37***	β_0 0.10 - β_1 7.68***
$EV_L > EV_R$	β_0 -0.90*** β_1 0.44***	β_0 -0.88*** β_1 0.54***	β_0 -1.23*** β_1 0.68***	β_0 -1.12*** β_1 0.73***	β_0 -1.01*** β_1 0.29***	β_0 -1.01*** β_1 0.26***	β_0 -0.32 - β_1 7.16***
$EV_R > EV_L$	β_0 1.22*** β_1 0.32***	β_0 1.19*** β_1 0.37***	β_0 0.83*** β_1 0.72***	β_0 0.78*** β_1 0.99***	β_0 1.12*** β_1 0.36***	β_0 1.12*** β_1 0.33***	β_0 0.55 - β_1 8.46***

Supplementary Table ST5. Weights for the logistic regression of choice and f_R. Regression weights (β_0, β_1) and significance (- n.s., *p<0.05, ***p<0.001) for results in Fig. 2B.

	offer1	*delay1*	*offer2*	*delay2*	*re-fixate*	*ch-hold*
EV_L	$1.44\ 10^{-151}$	$1.23\ 10^{-150}$	$1.14\ 10^{-149}$	$5.47\ 10^{-142}$	$1.98\ 10^{-149}$	0.513
m_L	$4.75\ 10^{-133}$	$1.02\ 10^{-133}$	$1.07\ 10^{-132}$	$1.33\ 10^{-129}$	$9.51\ 10^{-134}$	0.911
σ_L^2	$9.13\ 10^{-21}$	$1.63\ 10^{-20}$	$2.26\ 10^{-20}$	$2.42\ 10^{-20}$	$3.70\ 10^{-20}$	0.295
EV_R	$2.50\ 10^{-161}$	$4.73\ 10^{-160}$	$8.62\ 10^{-158}$	$3.03\ 10^{-150}$	$3.48\ 10^{-160}$	0.103
m_R	$1.12\ 10^{-151}$	$6.14\ 10^{-151}$	$1.25\ 10^{-147}$	$1.27\ 10^{-144}$	$2.67\ 10^{-151}$	0.911
σ_R^2	$7.95\ 10^{-23}$	$9.62\ 10^{-23}$	$6.70\ 10^{-23}$	$3.26\ 10^{-22}$	$8.74\ 10^{-23}$	0.227
s_{LR}	$2.37\ 10^{-5}$	$3.21\ 10^{-4}$	0.226	0.251	$3.32\ 10^{-4}$	0.875
f_R	$9.74\ 10^{-4}$	$3.70\ 10^{-2}$	$2.27\ 10^{-3}$	$4.29\ 10^{-10}$	$1.64\ 10^{-5}$	$1.27\ 10^{-34}$

	combined
EV_L	$2.37\ 10^{-152}$
m_L	$1.84\ 10^{-136}$
σ_L^2	$6.08\ 10^{-21}$
EV_R	$1.81\ 10^{-160}$
m_R	$2.56\ 10^{-149}$
σ_R^2	$1.71\ 10^{-23}$
s_{LR}	$9.90\ 10^{-4}$
f_R offer1	0.265
f_R delay1	0.064
f_R offer2	0.679
f_R delay2	0.029

Supplementary Table ST6. P-values for results in Fig. 2C (top) and Fig. 2D (left), FDR corrected.

	offer1	*delay1*	*offer2*	*delay2*	*ch-hold*
All trials	0.0013 **	$3.86\ 10^{-7}$ ***	0.2519 -	$9.25\ 10^{-4}$ ***	0.2519 -
Encoding EV_L	0.0017 **	$5.40\ 10^{-7}$ ***	0.1419 -	0.0392 *	0.0235 *
Encoding EV_R	0.2378 -	0.3638 -	$2.03\ 10^{-5}$ ***	$3.44\ 10^{-6}$ ***	$8.04\ 10^{-4}$ ***

Supplementary Table ST7. P-values for results in Fig. 3C and Fig. 3E, FDR corrected (significance: - n.s., *p<0.05, **p<0.01, ***p<0.001).

	offer1	*delay1*	*offer2*	*delay2*	*ch-hold*
SV_L vs SV_R	$3.424\ 10^{-6}$ ***	$6.528\ 10^{-9}$ ***	1.035 -	0.211 -	1.035 -
SV_R vs ch	0.052 -	0.094 -	0.013 -	0.127 -	$9.161\ 10^{-4}$ ***
SV_L vs ch	0.055 -	$2.835\ 10^{-4}$ ***	0.055 -	1.187 -	$2.402\ 10^{-5}$ ***
Encoding SV_L	$1.567\ 10^{-4}$ ***	$1.066\ 10^{-7}$ ***	0.347 -	0.004 **	0.004 **
Encoding SV_R	0.136 -	0.207 -	$3.162\ 10^{-5}$ ***	$2.877\ 10^{-6}$ ***	0.002 **
Encoding ch	0.755 -	0.755 -	1.287 -	0.773 -	0.755 -

Supplementary Table ST8. P-values for results in Fig. 5B and Fig. 5D, FDR corrected (significance: - n.s., *p<0.05, **p<0.01, ***p<0.001).

Supplementary Figure S1.

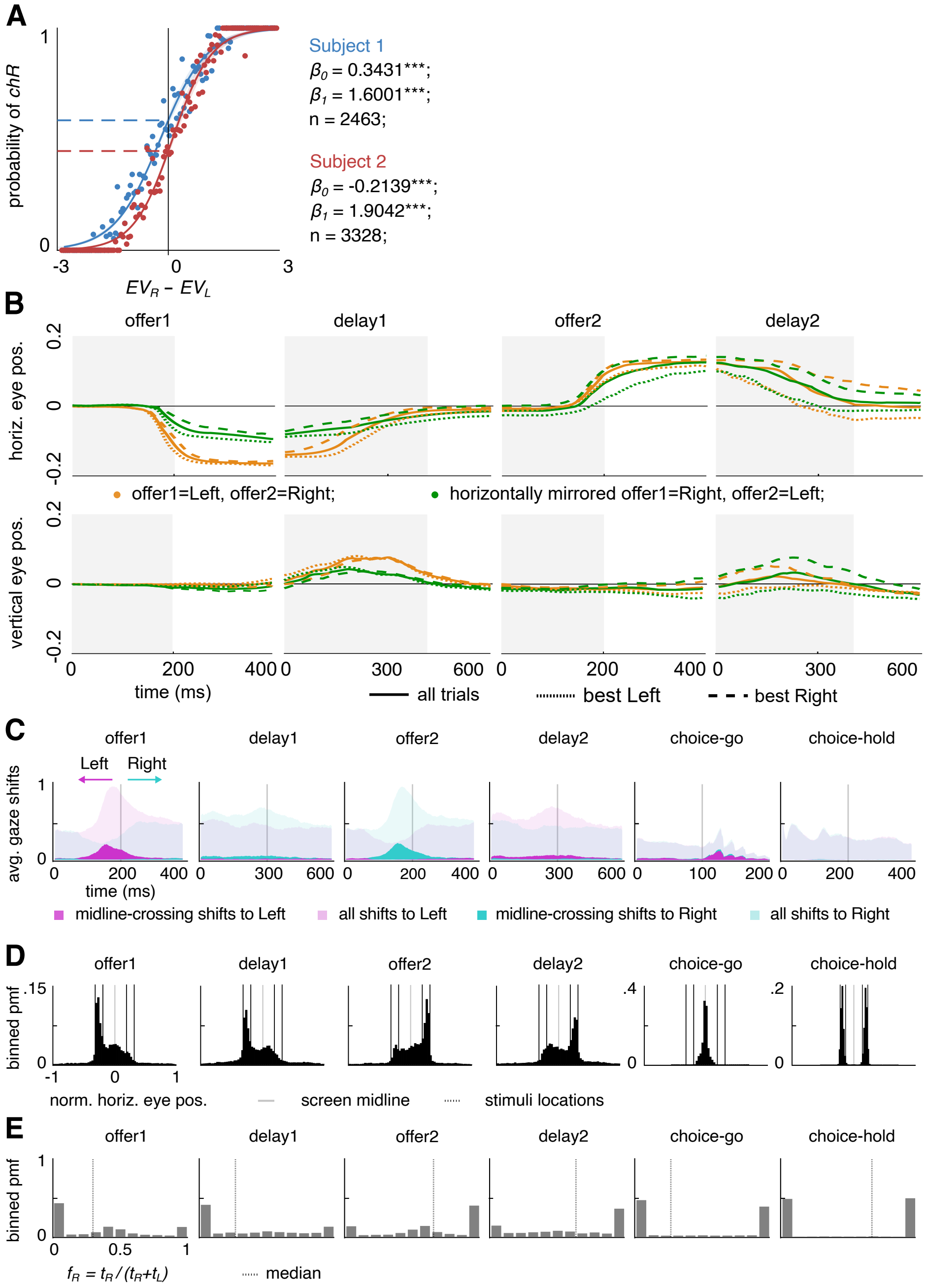

Supplementary Figure S1. Subject-specific performance, median gaze, and gaze shifts during task execution. A. Psychometric curves. Fits are made by using a logistic regression model of choice vs difference in expected values as in Fig.1B but for each subject. **B.** Top: median of horizontal eye position during task time execution (note: data for trials with first offer presented on the right screen side are horizontally mirrored); bottom: median of vertical eye position during task time. Solid lines include all trials, dotted lines only include trials where $EV_L > EV_R$, dashed lines only include trials where $EV_R > EV_L$. Data are combined from all trials in both subjects, in all experimental sessions. Data is separated based on whether the first offer is presented on the left screen side, or on the right side. **C.** Time histogram of gaze shifts detected as monotonic drifts in horizontal eye coordinate with duration at least 25ms. Midline crossing shifts are reported by solid areas. **D.** Binned probability mass function (pmf) of horizontal eye position (normalized to -1, +1) during task epochs. Solid vertical lines indicate screen midline, vertical dotted lines indicate stimuli positions. **E.** Binned pmf of f_R, computed in 10 ms bins, during task execution. Vertical, dotted lines indicate the median value.

Supplementary Figure S2.

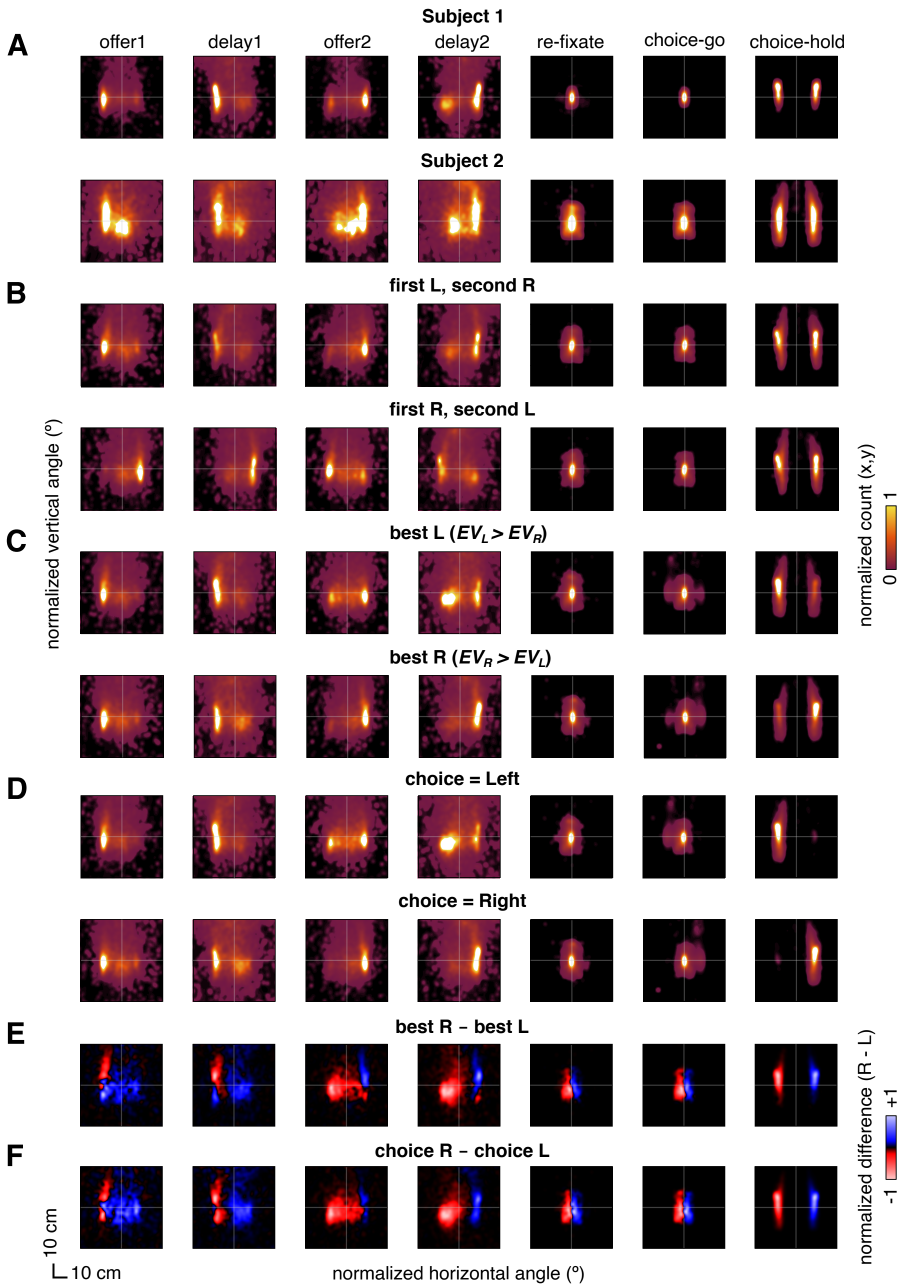

Supplementary Figure S2. Heatmaps of gaze during task execution at different task configurations. A. Distribution of eye position during all task epochs. Panels in the top row include all trials across sessions for subject 1, Panels in the bottom row include trials from sessions from subject 2. **B.** Same as A, but panels in the top row include data for all sessions and for both subjects for trials with first offer presented on the left screen side (second offer on right side), while panels in the bottom row include trials with first offer on the right screen side (second offer on left side). **C.** Same as B but showing trials where best offer is on the left (top) or on the right (bottom). **D.** Same as B, but panels in the top row include all trials across sessions in both subjects where the offer on the left screen side was chosen, while panels in the bottom row include trials with choice for the offer on the right screen side. **E.** Difference between the distribution of eye position with best offer is on the right side (blue, top in C) and best offer is on the left side (red, bottom in C). **F.** Difference between the distribution of eye position with choice for the right offer (blue, top row in D) and choice for the left offer (red, bottom row in D).

Supplementary Figure S3.

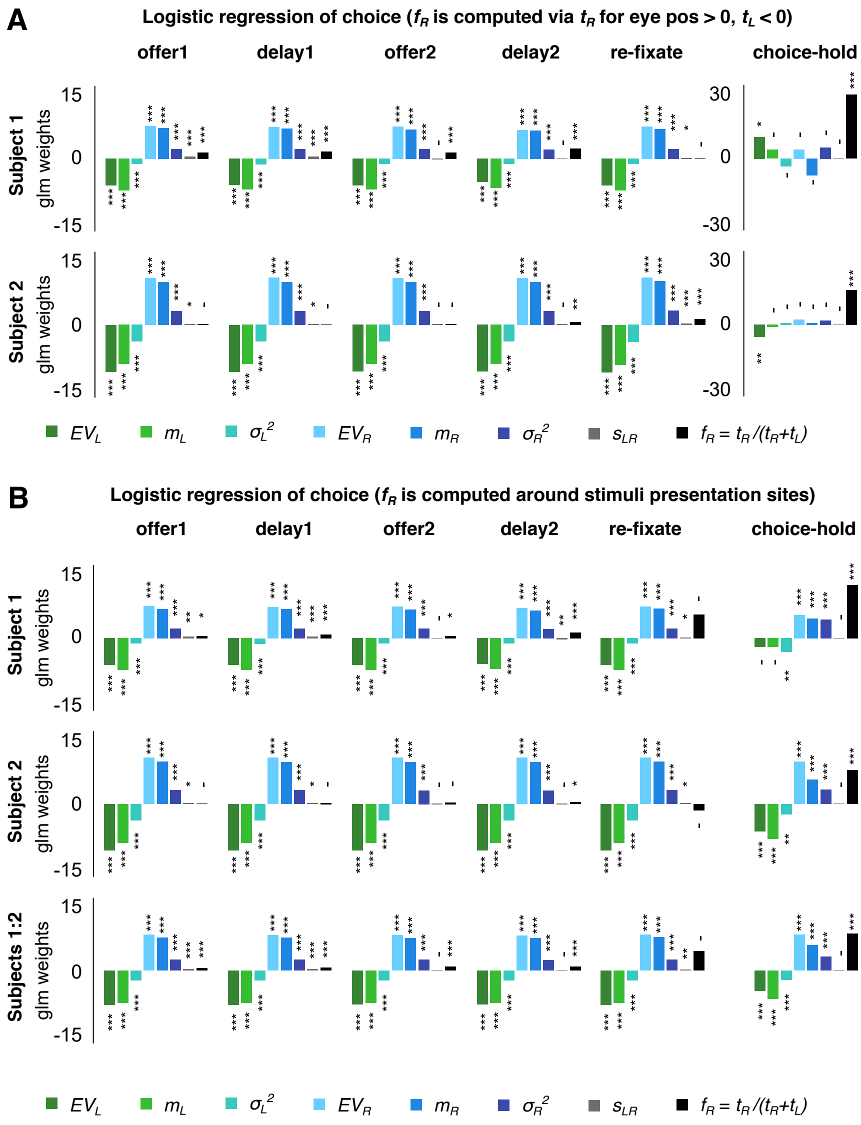

Supplementary Figure S3. Logistic regression of choice: subject-specific results and focus on stimuli locations. A. Same as in Fig. 2C but separated by subjects (top: subject 1; bottom: subject 2). **B.** Same as A but fraction of time refers to time looking L/R physical stimuli presentation sites rather than to L/R screen sides. Left presentation site: horizontal coordinates between -12.2±1.9cm; right site: +12.2±1.9cm.

Supplementary Figure S4.

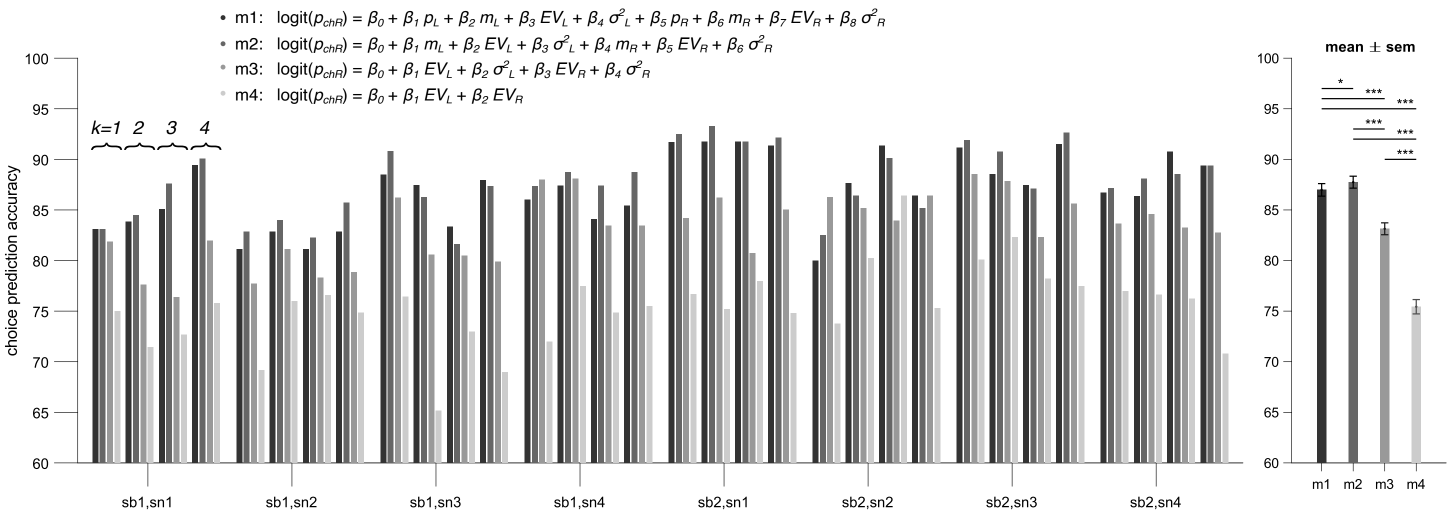

Supplementary Figure S4. Comparison of logistic regression models of the choice. Left: accuracy of prediction of choice by different logistic models of the choice. For data in each subject ($sb = 1, 2$), session ($sn = 1{:}4$) and for different models ($m1, m2, m3, m4$) we computed choice prediction and k-fold cross-validation over $k = 4$ disjoint data subsets. Right: mean ± SEM of the accuracy for each of the models, and mutual comparisons showing significant improvement as we progress from EV only model ($m4$); EV and σ^2 (***p<0.001 $m3$ vs $m4$); EV, σ^2 and magnitude regressors (***p<0.001 for $m1$, $m2$ vs $m3$, $m4$); and significant decrease when also including probabilities (*p<0.05 for $m2$ vs $m1$).

Supplementary Figure S5.

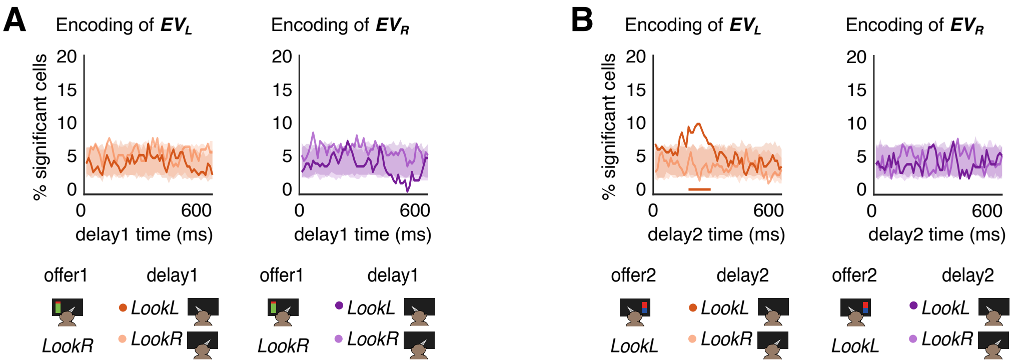

Supplementary Figure S5. Encoding of offer EVs during least common gaze patterns of subsequent offer/delay epoch pairs. A. Same as Fig. 3F, but *offer1 LookR*. **B.** Same as Fig. 3G, but *offer2 LookL*.

Supplementary Figure S6.

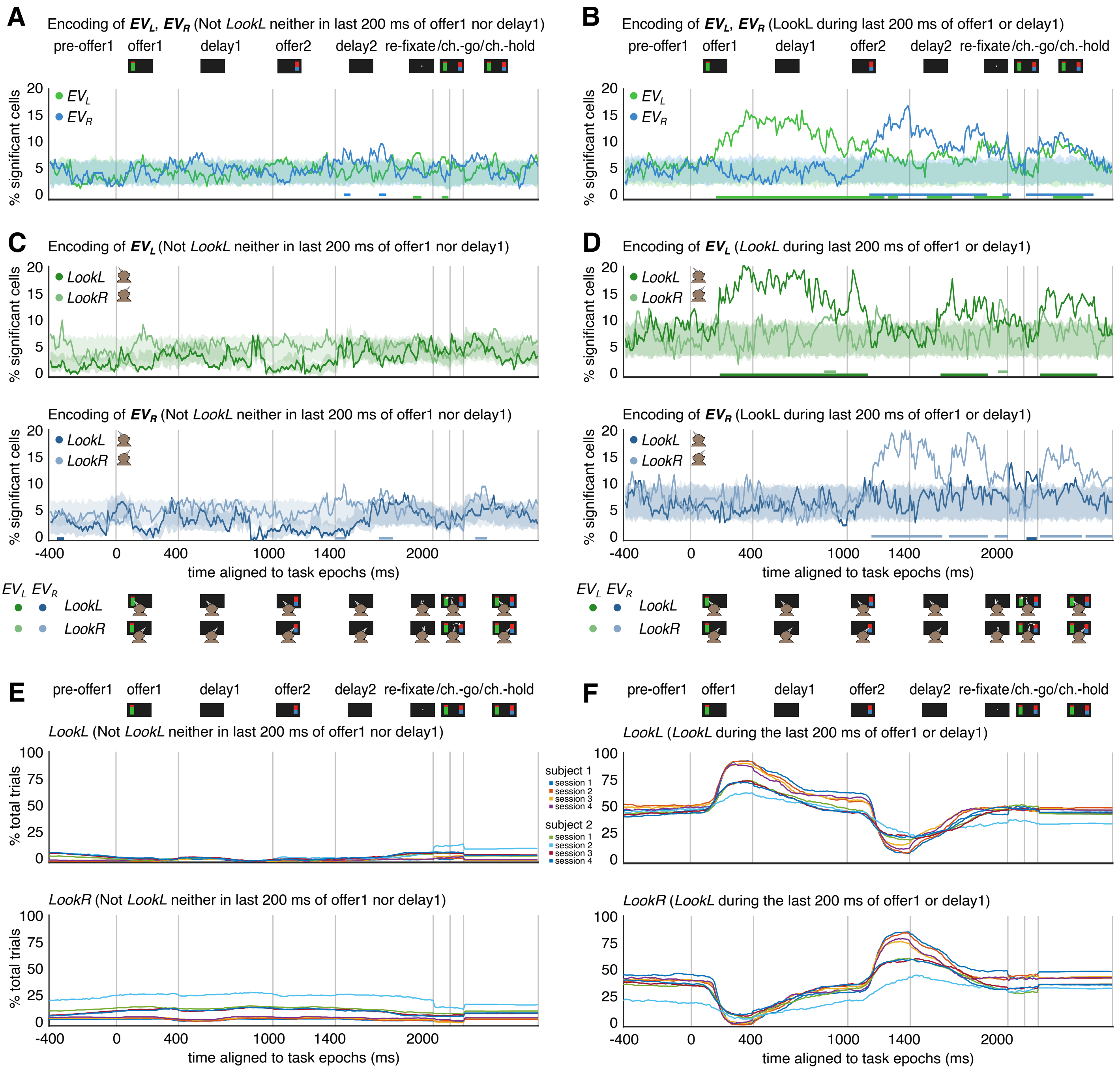

Supplementary Figure S6. Encoding of offer values: selecting trials where animals looked or did not look at the first offer during offer 1 or delay 1. Analysis of trials where subjects do not *LookL* during the last 200 ms of neither offer 1 nor delay 1 (A, C, E) and main analyses repeated for trials where subjects exclusively *LookL* in the last 200 ms of either offer1 or delay1 (B, D, F). Similar results are obtained by conditioning only on whether subjects *LookL* during the last 200 ms of *offer 1*, regardless of where they look at during *delay 1*. We use the stronger conditioning to avoid any possible residual memory of the first offer that could still be available during *delay 1*.

Supplementary Figure S7.

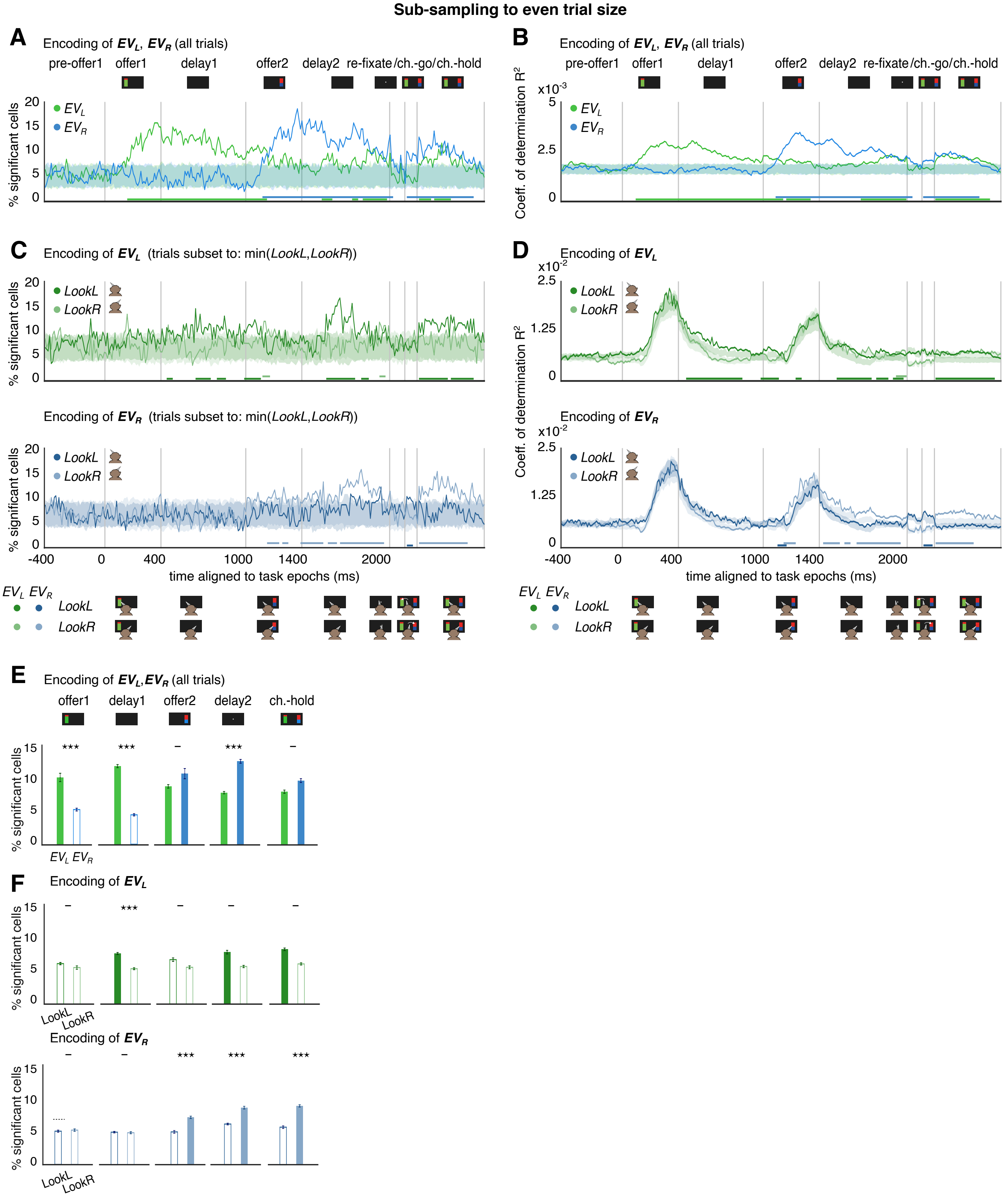

Supplementary Figure S7. Encoding of offer EVs by sub-sampling in trial pools of even size. A. Same as in Fig. 3B. **B.** Same as Supp. Fig. S8A. **C.** Same as Fig 3D, but for sub-sampled trial pools of even size. In each time bin, the number of trials for both *LookL* and *LookR* are matched in each session, and they are set to $n(t) =$ min (number of trials *LookL*, number of trials *LookR*). The number of subsets at each time bin is given by $m(t) = \lceil N/n(t) \rceil$, with N the total number of trials in each session. The average fraction of significant cells is computed for each subset, then fractions for all subsets are averaged. **D.** Average coefficients of determination R^2 for sub-sampled results in C (to compare with Supp. Fig. S12C). Overall, sub-sampled results qualitatively match main results in Fig. 3, but they are weaker due to the lower number of trials used. **E.** Same as in Fig 3C. **F.** Same as in Fig. 3E, but for sub-sampled trial pools of even size.

Supplementary Figure S8.

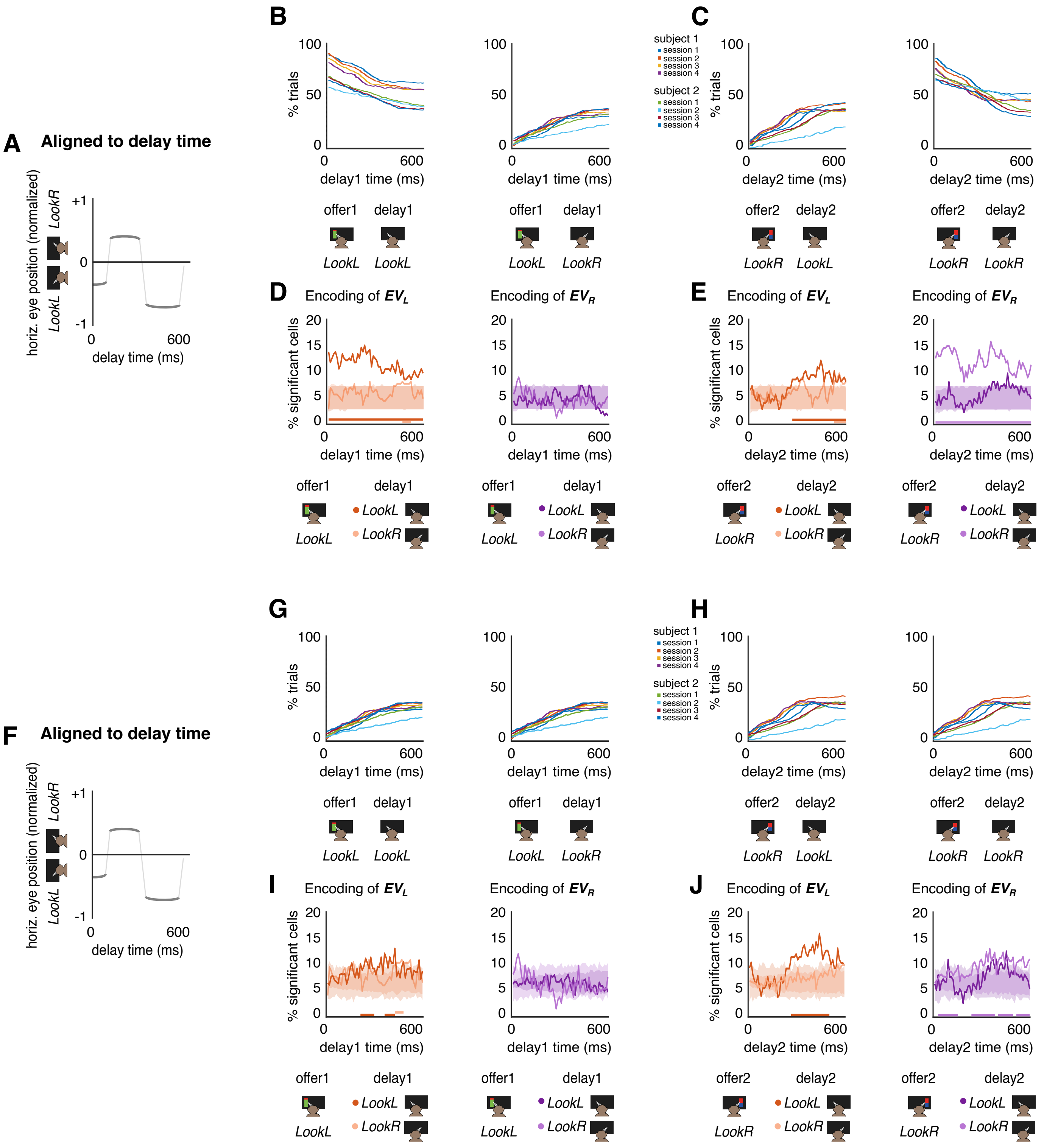

Supplementary Figure S8. Encoding of offer values at delay epochs: trials availability and subsampling to trial pools of even size. A, F. Sketch of settings used for computation, to be compared to Supp. Fig. S14 A, F. **B-C.** Fractions of trials available in Fig. 3F-G. **D-E.** Same as Fig. 3F-G. **F-J.** Same as Fig. 3F-G but subsampled to equally sized subsets of available trials, shown in G-H.

Supplementary Figure S9.

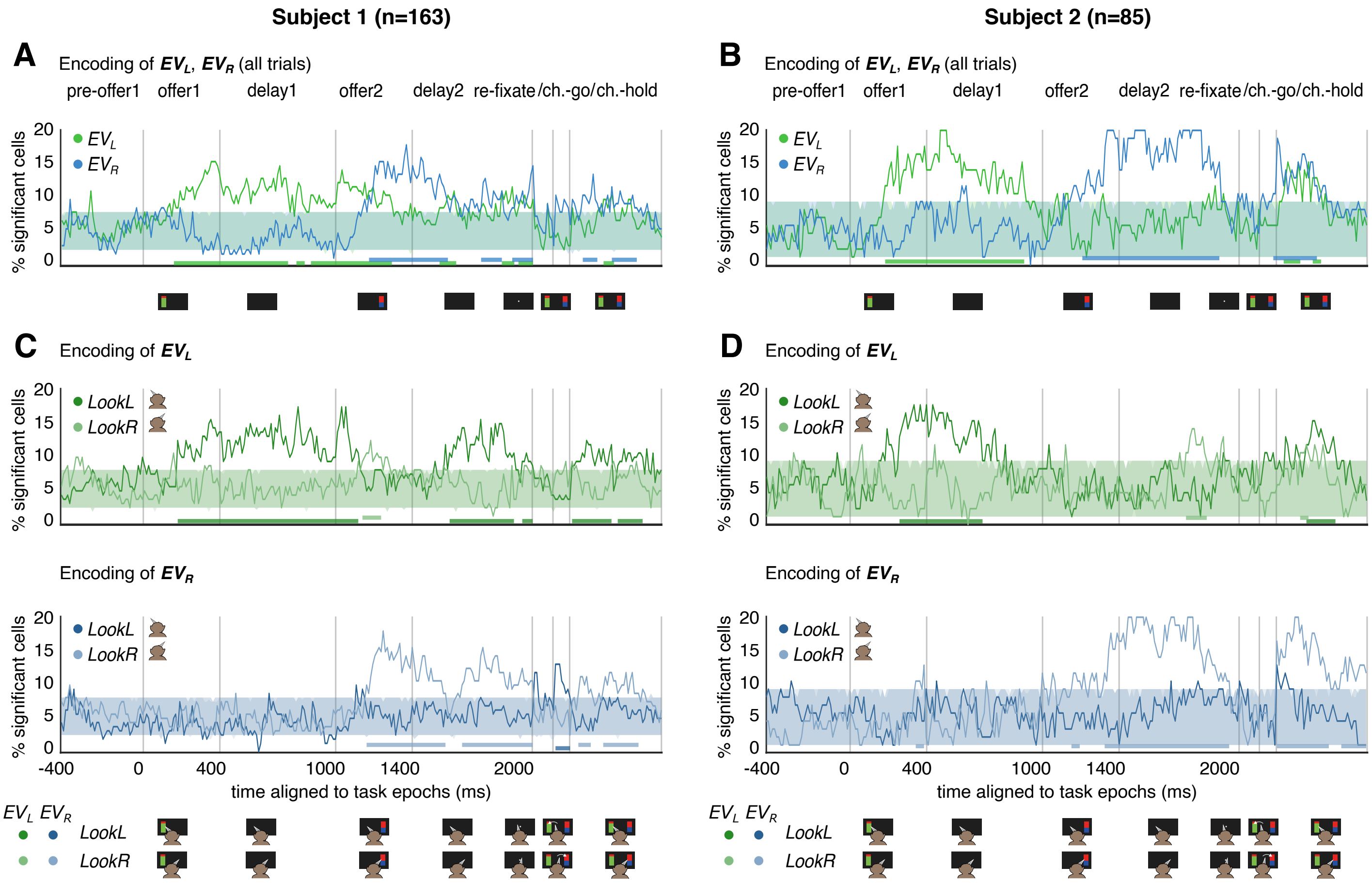

Supplementary Figure S9. Encoding of offer EVs: subject-specific results. A, B. Same as Fig. 3B for subject 1 (A), subject 2 (B). **C, D.** Same as Fig. 3D for subject 1 (C), subject 2 (D).

Supplementary Figure S10.

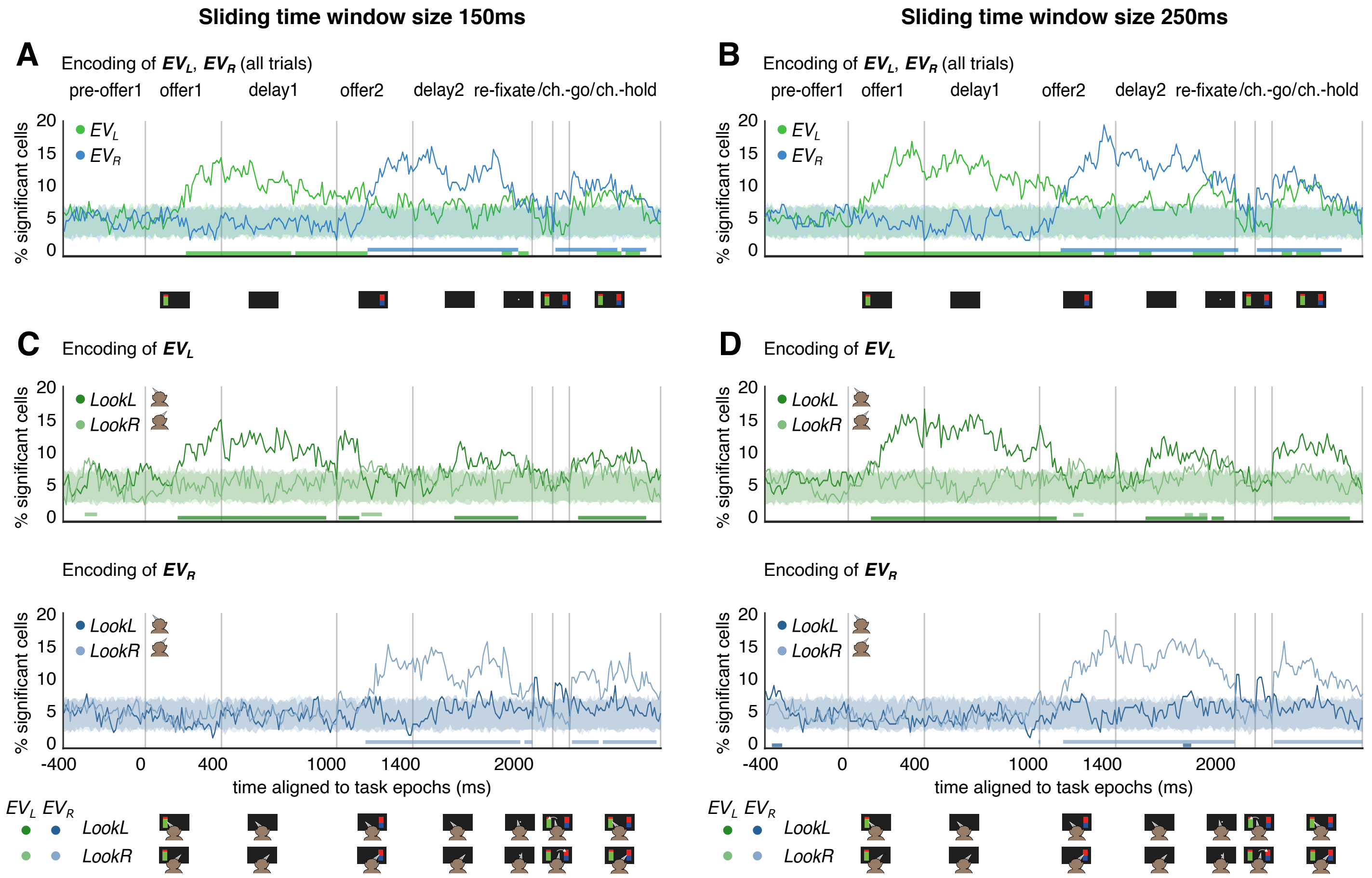

Supplementary Figure S10. Encoding of offer EVs: longer/shorter spike rate time windows. A, C. Same as Fig. 3 B, D but for sliding time windows of size 150 ms. **B, D.** Same as Fig. 3 B, D. but for sliding time windows of size 250 ms.

Supplementary Figure S11.

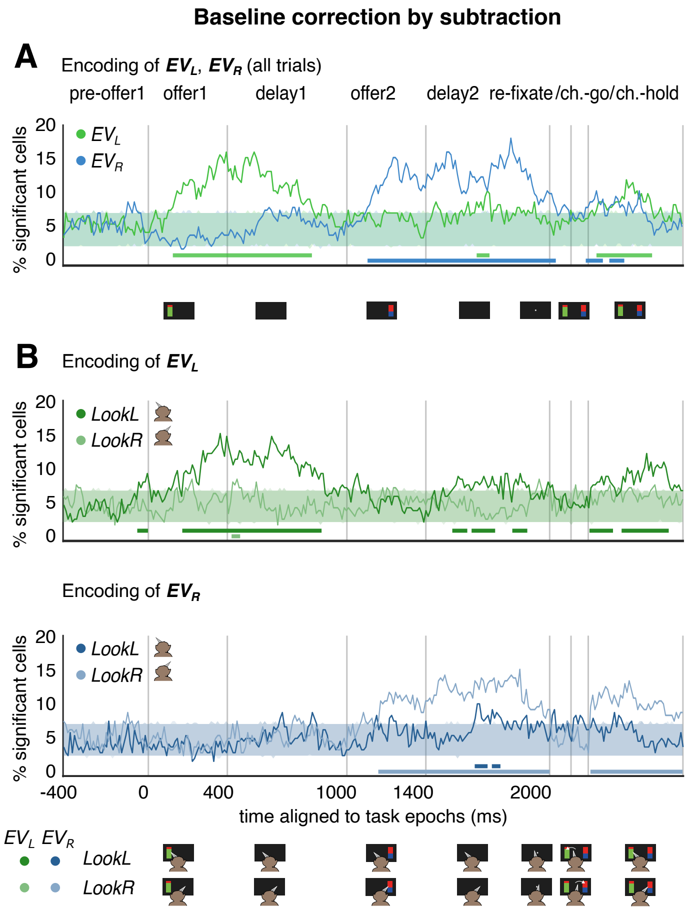

Supplementary Figure S11. Encoding of offer EVs with baseline correction. A. Same as Fig. 3B but for baseline correction by subtraction of spike rate in each time bin by time-averaged spike rate in *pre-offer1* epoch time. **B.** Same as Fig. 3D but for baseline correction as in A.

Supplementary Figure S12.

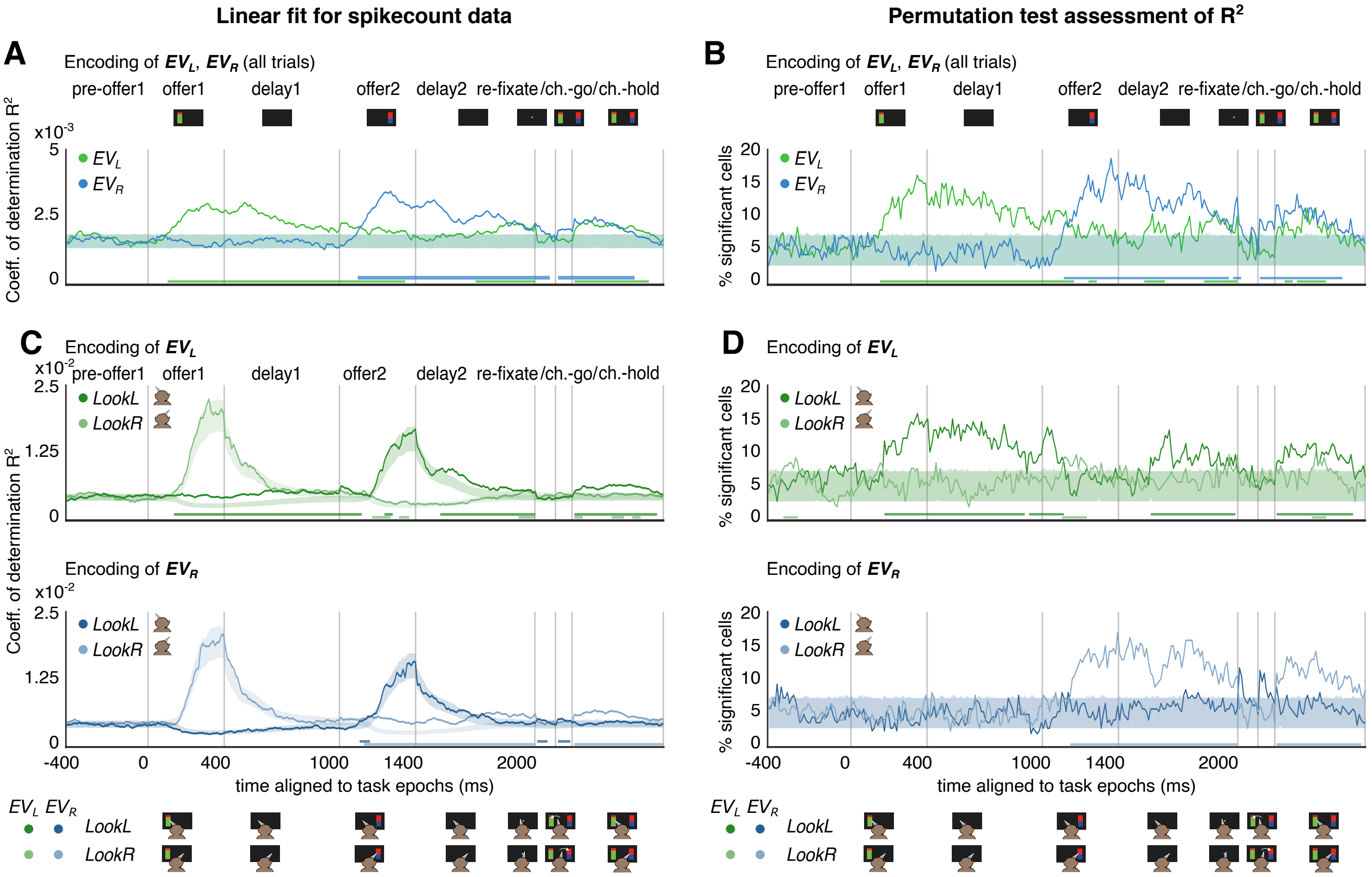

Supplementary Figure S12. Encoding of offer EVs: coefficient of determination and significance assessment via permutation tests. A. Coefficient of determination R^2 for the results in Fig. 3B. Top: R^2 for the linear encoding of EV_L (green) and EV_R (blue) throughout the trial. Shaded areas report 5-to-95th percentile of R^2 distribution computed by n=1000 independent shuffles of the trial order for both EVs. Colored lines at the bottom of the panel show time bins with R^2 above 95th percentile (run length assessment). The changes in magnitude of shaded areas are due to the different numbers of trials available and used to fit linear models and to run the permutation tests. Note that *LookL* trial availability is higher in *offer1*, *delay1* while *LookR* trial availability is higher in *offer 2*, *delay 2*. **B.** Assessment of the significance of R^2 in panel A via permutation tests. **C.** Same as A but focusing on EV_L (top) and EV_R (bottom), comparing in each time bin results for trials where subjects mostly *LookL* (green or blue, respectively) vs *LookR* (light green or light blue, respectively). **D.** Same as C, but for results in panel B.

Supplementary Figure S13.

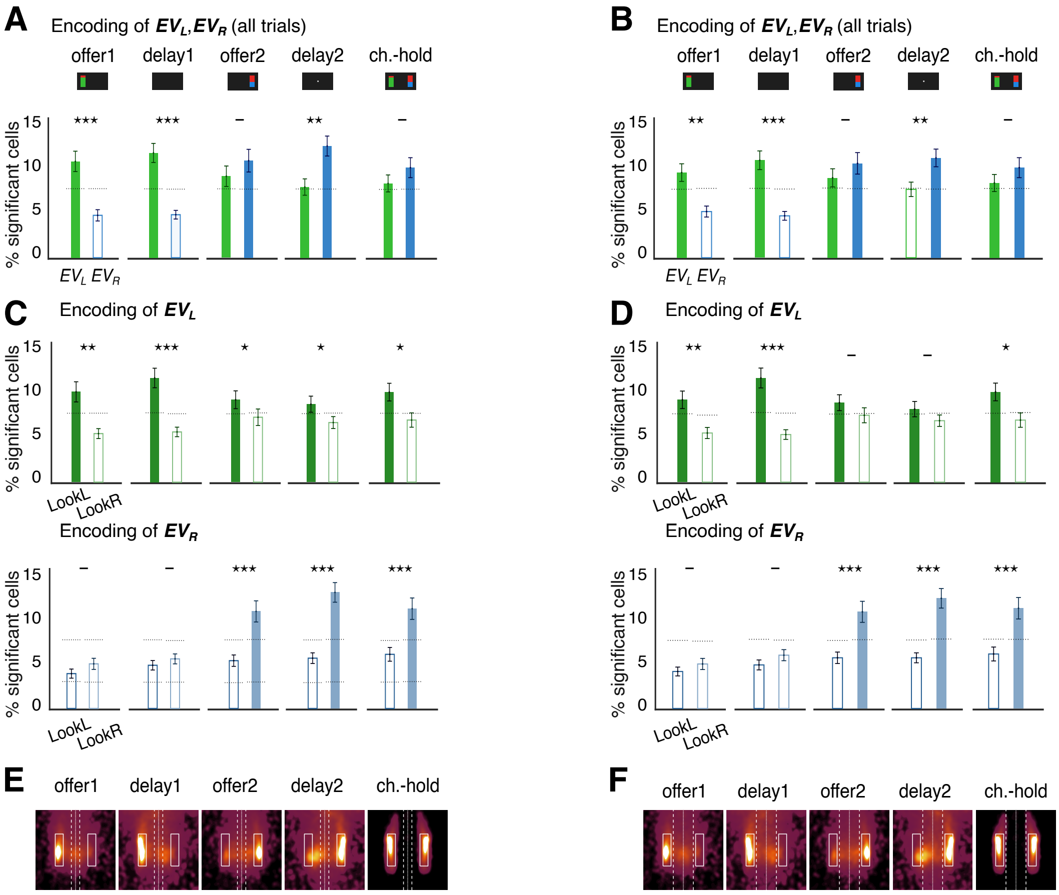

Supplementary Figure S13. Encoding of offer values: removing data close to the vertical screen midline. Same as Fig. 3C, 3E but removing data within 4 cm (A, C) and 9.5 cm (B, D) from screen midline center. Heatmaps of gaze position during task execution show data removal thresholds (at 4 cm, E; or at 9.5 cm, F) from screen midline as white dashed lines. In Panel D, delay 2 epoch, significance of reactivation is lost, most likely because many trials with gaze to the left are removed in this more stringent condition (F, delay 2 epoch).

Supplementary Figure S14.

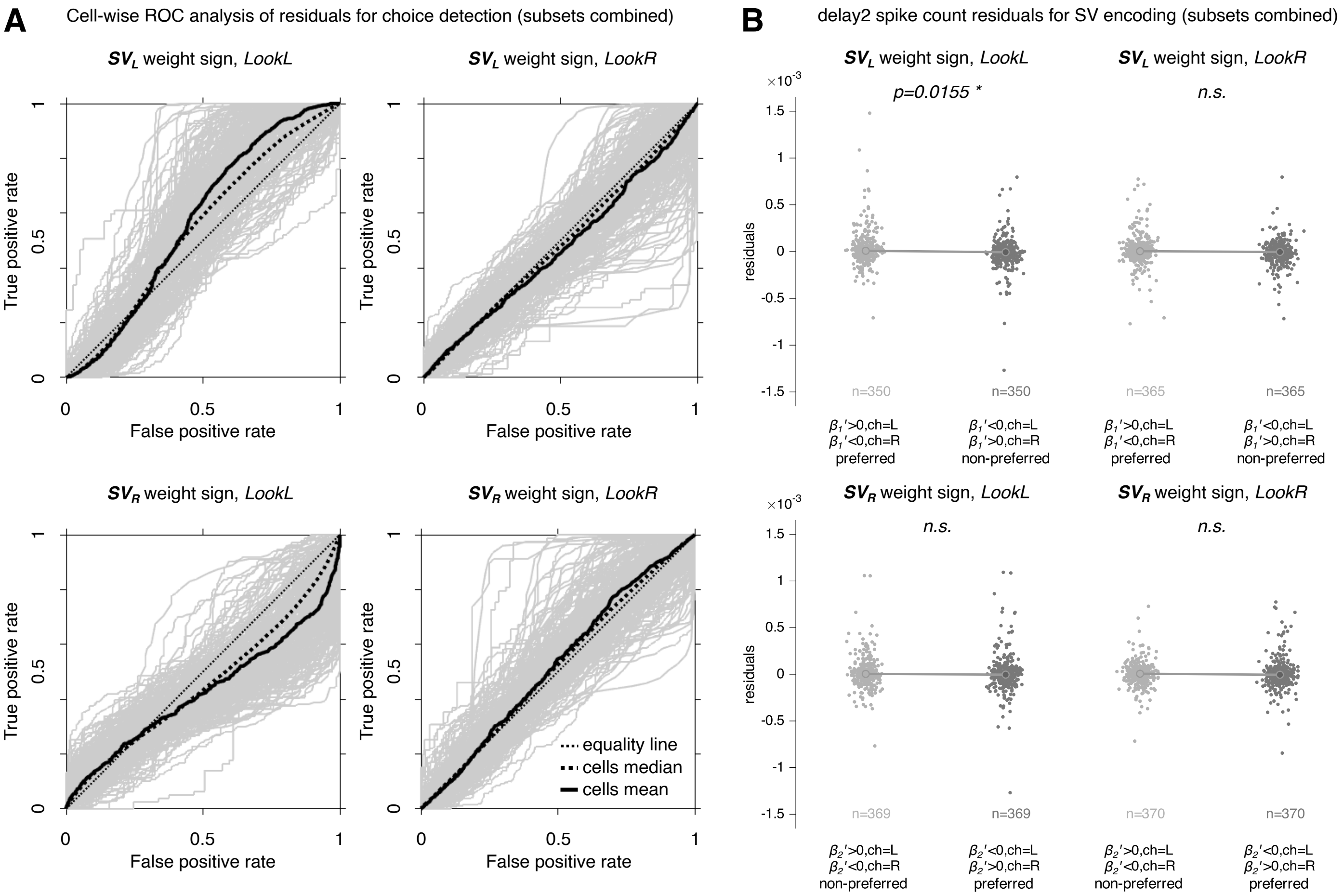

Supplementary Figure S14. Comparison of spike count regression residuals for different SV tuning signs and choices. A. Cell-wise ROC analysis of residuals magnitude. Area Under the Curve (AUC; SV_L, *LookL*: median $AUC = 0.56$; SV_L, *LookR*: median $AUC = 0.48$; SV_R, *LookL*: median $AUC = 0.44$; SV_R, *LookR*: median $AUC = 0.52$) respectively match the median of *LookL/LookR* CPs in Fig. 5E-F. The ROC for only significant modulations (as assessed by F-statistics on β_1' or $\beta_2' \neq 0, p < 0.05$) are not reported as they do not show appreciable difference compared to ROC including all modulations in A., but AUC showed slightly larger median differences (significant SV_L modulation: *LookL* median $AUC = 0.60$, *LookR* median $AUC = 0.51$; significant SV_R modulation: *LookL* median $AUC = 0.43$, *LookR* median $AUC = 0.54$). **B.** Least squares fit residuals for the simplified linear model $\eta = \beta_0' + \beta_1' SV_L + \beta_2' SV_R$ for different choices (*preferred* and *non-preferred*) at *delay 2*. Data are pooled as labelled in x-axis and averaged across time and trials for each cell. The analysis is applied separately to SV_L (top) and SV_R (bottom) weights signs in trials where subjects *LookL* (left) or *LookR* (right), compared via one-tailed paired Wilcoxon signed rank tests (testing *preferred* > *non-preferred*; *p<0.05).

Supplementary Figure S15.

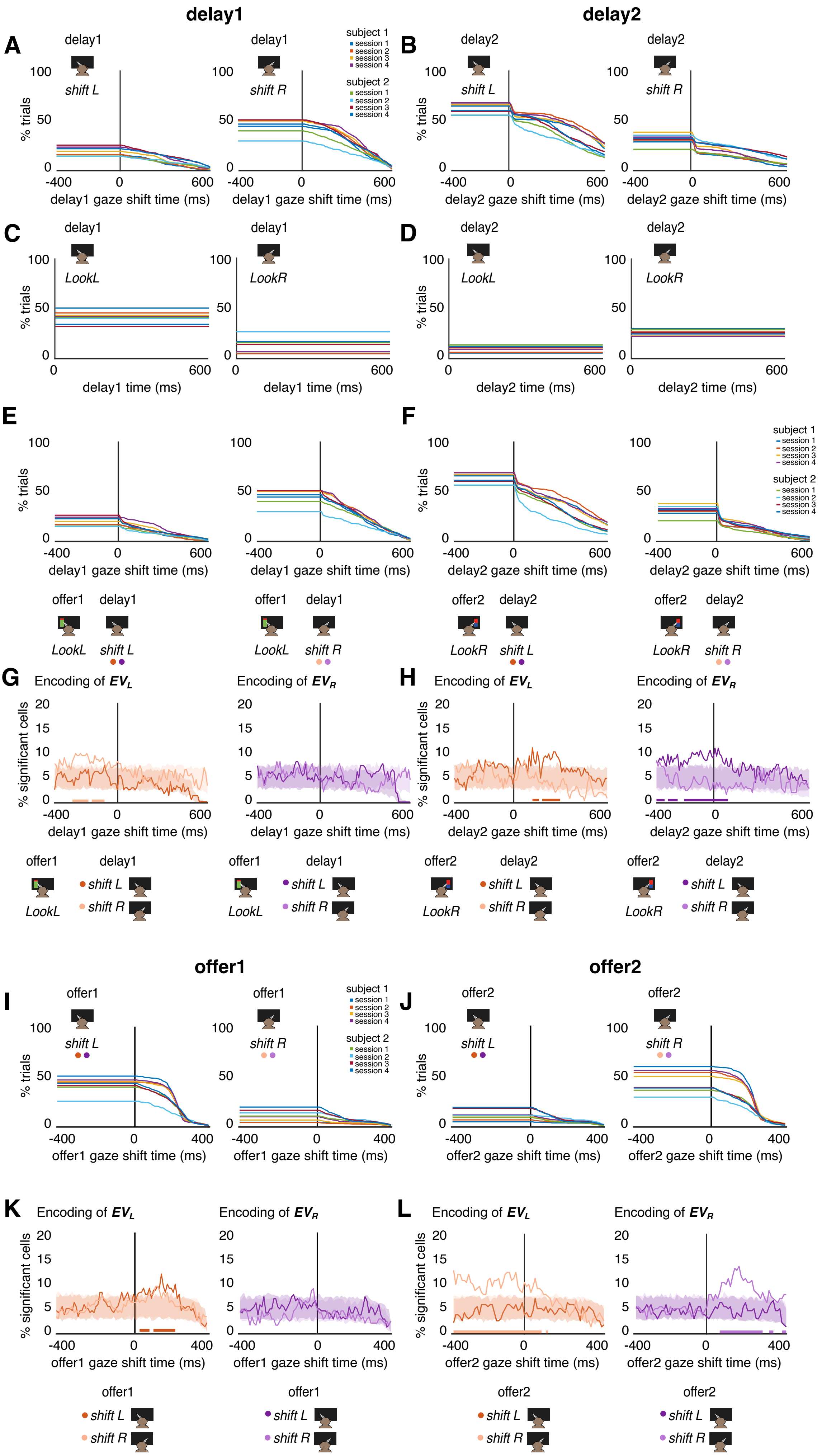

Supplementary Figure S15. Encoding of offer values at offer epochs aligned to midline-crossing gaze shifts. A-D. Fractions of trials available in the respective configurations of Fig. 4A-E. **E-H.** Same as A-B and Fig. 4B-C, but conditioning pre-shift gaze (200 ms prior to shift initiation) on the side of offer presentation (left in *offer 1*, right in *offer 2*). **I-L.** Same as A-B and Fig. 4B-C but aligned to the occurrence of first midline-crossing gaze shift in offer epochs. Encoding of either offer increases soon after gaze is shifted towards the respective presentation screen side (D, dark orange; E, light purple). Conversely, the encoding of left offer lowers whenever subjects shifted gaze away from left screen side (E, light orange). In all other cases we do not find significant encoding.
